# Exosome-Depleted Excretory-Secretory Products of the Fourth-Stage Larval *Angiostrongylus cantonensis* Promotes Alternative Activation of Macrophages Through Metabolic Reprogramming by the PI3K-Akt Pathway

**DOI:** 10.3389/fimmu.2021.685984

**Published:** 2021-07-23

**Authors:** Shuo Wan, Xiaoqiang Sun, Wenyan Tang, Lifu Wang, Zhongdao Wu, Xi Sun

**Affiliations:** ^1^ Department of Parasitology of Zhongshan School of Medicine, Sun Yat-sen University, Guangzhou, China; ^2^ Key Laboratory of Tropical Disease Control (SYSU), Ministry of Education, Guangzhou, China; ^3^ Provincial Engineering Technology Research Center for Biological Vector Control, Guangzhou, China; ^4^ The First Affiliated Hospital, Jinan University, Guangzhou, China; ^5^ Zhongshan School of Mathematics, Sun Yat-Sen University, Guangzhou, China; ^6^ Department of Neonatology, Guangzhou Women and Children’s Medical Center, Guangzhou Medical University, Guangzhou, China

**Keywords:** *Angiostrongylus cantonensis*, exosome-depleted excretory-secretory products, N-linked glycoproteins, macrophage polarization, mechanism

## Abstract

*Angiostrongylus cantonensis* (AC), which parasitizes in the brain of the non-permissive host, such as mouse and human, is an etiologic agent of eosinophilic meningitis. Excretory-secretory (ES) products play an important role in the interaction between parasites and hosts’ immune responses. Inflammatory macrophages are responsible for eosinophilic meningitis induced by AC, and the soluble antigens of *Angiostrongylus cantonensis* fourth stage larva (AC L4), a mimic of dead AC L4, aggravate eosinophilic meningitis in AC-infected mice model *via* promoting alternative activation of macrophages. In this study, we investigated the key molecules in the ES products of AC L4 on macrophages and observed the relationship between metabolic reprogramming and the PI3K-Akt pathway. First, a co-culture system of macrophage and AC L4 was established to define the role of AC L4 ES products on macrophage polarization. Then, AC L4 exosome and exosome-depleted excretory-secretory products (exofree) were separated from AC L4 ES products using differential centrifugation, and their distinct roles on macrophage polarization were confirmed using qPCR and ELISA experiments. Moreover, AC L4 exofree induced alternative activation of macrophages, which is partially associated with metabolic reprogramming by the PI3K-Akt pathway. Next, lectin blot and deglycosylation assay were done, suggesting the key role of N-linked glycoproteins in exofree. Then, glycoproteomic analysis of exofree and RNA-seq analysis of exofree-treated macrophage were performed. Bi-layer PPI network analysis based on these results identified macrophage-related protein Hexa as a key molecule in inducing alternative activation of macrophages. Our results indicate a great value for research of helminth-derived immunoregulatory molecules, which might contribute to drug development for immune-related diseases.

## Introduction

Angiostronglyiasis is a serious foodborne parasitic disease caused by infection of AC, which parasitizes in the brain of the non-permissive host mouse and human and leads to serious eosinophilic meningitis; however, the underlying mechanism remains poorly understood. Microglia/macrophages are major immune cells involved in directing host inflammatory and immune processes, which are extremely important in helminth infections. Microglia, brain-resident macrophages, are activated in AC-infected mice; however, minocycline (1), a specific inhibitor of microglia activation, failed to reverse the brain inflammation. In our previous results, Chi3l3, an eosinophil-related protein, was found to increase sharply after AC infection, and monocyte-derived alternative macrophages were confirmed as the main source of Chi3l3 (2), which indicated that M2 macrophage may be involved in the pathogenesis of Angiostrongyliasis.

It is widely assumed that helminth could modulate host immune responses through its ES products, including proteins, glycans (3, 4), and exosomes (5–7). *Fasciola hepatica*–derived Fh12 and Fh15 could significantly promote M2 polarization of macrophages and inhibit TLR2-, TLR5-, TLR8-induced M1 polarization of macrophages (8). *Acanthocheilonema viteae*–derived AvCystatin was shown to induce IL-10 production of macrophages and inhibit T cell proliferation and restore allergic airway inflammation (9). Moreover, AvCystatin could significantly upregulate PD-L1 and PD-L2 levels in macrophages and protect against mucosal inflammation (10). MIF homologs in *Brugia malayi* could promote IL-4-induced macrophage alternative polarization through upregulating IL-4Rα (11). While *Trichinella spiralis* rTsP53 could induce significant alternative activation of macrophages *via* STAT6 but not IL-4Rα *in vitro* (12). *Fasciola hepatica*–derived FhHDM-1 was shown to bind directly to LPS and protect mice against LPS-induced inflammation by reducing inflammatory mediators from macrophages (13). *S. japonicum* extracellular vesicle miR-125b and bantam miRNAs could be internalized by macrophages and modulate cell proliferation and TNF-α production, which contributes to parasite survival (14). Meanwhile, numerous studies have highlighted the importance of glycans as their interactions with the host immune system, as they were found on the surfaces of helminths and within their ES products (15, 16), which might interact with host glycan-binding proteins, such as C-type lectin receptors and galectins, shaping innate and adaptive immune responses upon infection. High-mannose-type glycans were identified in *Taenia solium metacestodes* (17). Glycan determinants of *Trichuris suis* soluble products modulate LPS-induced activation of human DCs (18). SEA and Omega-1 from *Schistosoma manosoni* were also proved to drive DCs for Th2 induction (19) and modulated the progression of experimental autoimmune encephalomyelitis (20). Thus, identification of the important compound or protein that has the function of modulating host immune responses is of great significance for understanding the interaction of parasite and host.

Recently, the functions of exosomes in parasite-host interactions are given considerable attention. Exosomes are nanosized membrane-bound extracellular vesicles with a diameter of 50–200 nm which are released from most cell types (21). They contain different biomolecules, including proteins, microRNAs, lipids, glycans, etc., performing biological functions, particularly in cell-to-cell communication. Recently, studies have found that exosomes can be used by parasites to deliver molecules and modulate host immune response (22).

Our previous study suggested that the soluble antigens (sAg) of AC L4, a mimic of dead AC L4, aggravate eosinophilic meningitis in the AC-infected mice model, *via* a Chi3l3-IL-13 positive feedback loop (2). However, the key compound or protein in the ES products of live AC L4 is not clear. In this study, the ES products of live AC L4 were separated into exosomes and exofree. Interestingly, our results suggested that AC L4 exofree, but not exosome, could induce a significant M2 polarization of macrophages through metabolic reprogramming by the PI3K-Akt pathway. Considering that exofree of N-linked glycosylated analogs failed to induce M2 polarization of macrophages, mass spectrometry analysis was performed to identify proteins with N-glycosylation sites in AC L4 exofree. Proteins with N-glycosylation sites in AC L4 exofree were identified using mass spectrometry analysis. Furthermore, RNA-seq analysis of exofree-treated macrophages was performed. Bi-layer PPI network analysis based on the results above supports that macrophage-related protein Hexa in exofree is a key molecule in inducing alternative activation of macrophages.

Collectively, our results suggest that AC L4 might modulate macrophage function through its ES products. Especially, N-linked glycoproteins from exosome-depleted ES products of AC L4 could induce significant alternative activation of macrophages through metabolic reprogramming by the PI3K-Akt pathway. Our results support that Hexa, a macrophage-related protein, might be the key molecule in exofree in inducing macrophage polarization, which might play a key role in directing host CNS inflammation in AC infection. The results of this study indicate a great value for research of helminth-derived immunoregulatory molecules, which might contribute to drug development for immune-related diseases.

## Materials and Methods

### The Animal Studies

Male Sprague Dawley (SD) Rat (specific pathogen-free, SPF) aged 6 weeks (weighing 100–120 g) were purchased from the Guangdong Medical Laboratory Animal Center. The animal studies were approved by the Medical Research Ethics Committee of Sun Yat-sen University and conformed to the Chinese National Institute of Health Guide for the Care (No SYSU-IACUC-2019-535). The rats were group-housed in ventilated cages in a temperature-control room (25°C) and were fed standard mouse chow. Each SD rat was infected with 600 AC L3 larvae (the third stage larvae) to collect the AC L4 (the fourth stage larvae) for *in vitro* studies.

### Preparation of Macrophages and Reagents

Bone marrow leukocytes were resuspended at 1.5×10^7^ cells/10 ml in macrophage media (DMEM high glucose with 10% heat-inactivated (56°C, 30 min) FBS, 100 U/ml of penicillin-streptomycin, supplemented with 20 ng/ml M-CSF) and then plated in 10 cm non-TC treated culture dishes. BMDMs were cultured for 7 days, with a media change on D3. BMDMs were detached from the dish using warm 0.05% trypsin and cultured in tissue culture plates for functional assay. The following reagents and working concentration were used in this study: recombinant murine M-CSF (20 ng/ml, 315-02; Peprotech), recombinant mouse IL-4 (10 ng/ml; 214-14-5, Peprotech), recombinant mouse IL-13 (10 ng/ml; 413-ML-025/CF, R&D Systems), Ly294002 (10 μM, S1737-1mg, Beyotime), Wortmannin (100 nM, S1952, Beyotime), GW9662 (1 μg/ml, S2915, selleckchem), Leflunomide (100 μM, S1247, selleckchem), Affi-Gel Hz Immunoaffinity Kit (#1536060, Biorad), PNGase F (500 units/μl, P0704S, New England Biolabs). For the BMDM functional experiment, AC L4 exosomes working concentration was 25 μg/ml, and AC L4 exofree working concentration was 4.4, 44, and 440 μg/ml. LPS working concentration was 0.05 μg/ml. sAg working concentration was 25 μg/ml. IL-4/IL-13 working concentration was 10 ng/ml.

### Isolation of AC L4 Exofree and AC L4 Exosome

AC L4 were collected from rat brains at 21 days post-infection and cultured in concentration up to 200 worms/ml with serum-free media [DMEM high glucose, 100 U/ml of penicillin-streptomycin] *in vitro* in 37°C. The ES products were collected every day for a maximum of 1 week. AC L4 exosome and exofree (extracellular vesicle–deleted exosome) were purified by differential centrifugation according to the recommended protocol (23). Briefly, larvae and pellets were removed by low-speed spinning at 300 × g (10 min at 4°C), followed by 2,000 × g for 10 min at 4°C, 10,000 × g for 30 min at 4°C to deplete residual debris, and then 120,000 × g for 120 min at 4°C to gather AC L4 exofree and AC L4 exosome. AC L4 exosomes were resuspended with PBS and stored at 4°C for short-term (1~7 days) or −80°C for long-term storage. AC L4 exofree without N−glycosylated analogs was obtained as previously described (24). Briefly, AC L4 exofree was first desalted with spin desalting column after incubating in boiling water for 10 min. Then, the desalted solutions were resuspended with oxidation buffer. Next, 50 mM NaIO4 was added to the solutions, and the reactions were kept in the dark at room temperature for 1 h. Then, 100 mM Na2S2O3 was added to the oxidized solutions to quench the oxidation reaction. The solution was added to the prewashed hydrazide resins. The coupling reaction was carried out with gentle shaking at 4°C overnight. Then, 1,000 units of PNGase F in PBS were added to the resins, and the released supernatant was collected into a dialysis bag. The supernatant was dialyzed in PBS for 6 h overnight at 4°C. The supernatant was concentrated and stored as deglycosylated AC L4 exofree. The same volume of exofree and deglycosylated AC exofree was used for BMDM functional assay in **Figures 5L–M**.

### Functional Experiments of Macrophages

BMDMs and freshly isolated live AC L4 (10 worms per well) was co-cultured for 12 h, and Chi3l3 level in the culture medium was measured using ELISA method. IL-13 working concentration was 10 ng/ml in this experiment. OCR and ECAR were monitored consecutively with a Seahorse Bioscience extracellular flux analyzer (XF24, Seahorse Bioscience) as described previously. Briefly, BMDMs were cultured in 24-well seahorse cell culture plate, 15,000–25,000 cells per well in 0.5 ml medium (2), stimulated with PBS, LPS (0.05 μg/ml), IL-13 (10 ng/ml), AC L4 exosome (25 μg/ml), and AC L4 exofree (44 μg/ml) for 24 h in a 37°C incubator. Then, cells were immersed in 500 μl specified medium following two wash steps with specified medium and incubated in an incubator without CO2 for 1 h before the measurements. The OCR and ECAR were then measured in a typical 8-min cycle of mix (2–4 min), dwell (2 min), and measure (2–4 min) as recommended by Seahorse Bioscience. Then 1 μM oligomycin, 1.0 μM FCCP, 0.5 μM rotenone + 0.5 μM antimycin, 10 mM glucose, 1.0 μM oligomycin, and 50 mM 2-deoxyglucose (2-DG) were used in this experiment. For qPCR and ELISA assay, BMDMs were cultured in 12-well cell culture plate, 10^6^ cells per well in 0.5 ml medium, stimulated with IL-13 (10 ng/ml), IL-4 (10 ng/ml), AC L4 exofree (4.4, 44, 440 μg/ml) for 24 h. For CCK8 assay, BMDMs were cultured in 96-well tissue culture plate, 5 × 10^4^ cells per well in 0.2 ml medium, stimulated with IL-13 (10 ng/ml), AC L4 exofree (4.4, 44, 440 μg/ml) for 6, 12, 24, 48, and 72h. Especially, to define the key molecule from exofree, ultracentrifugal filters were used for exofree fractionation, and the equal volume (200 μl) of fractionated AC L4 exofree was used for BMDM stimulation (24 h) and the qPCR analysis. Oligomycin (1 μM, 495455-10MG, Sigma-Aldrich), FCCP (50 nM, C2920-10MG, Sigma-Aldrich), 2-DG (10 mM, D8375-10MG, Sigma-Aldrich) were used in this experiment. All the experiments consist of quadruplicate biological replicates.

### Exosome Characterization and Exosome Uptake Assay

The concentration of exosomes (number/ml) and size distribution (in nanometer) of AC L4 were analyzed using IZON qNano particle analyzer (Izon Science Ltd.) equipped with fast video capture and Nanoparticle Tracking Analysis software. The samples were captured for 60 s at room temperature. Each sample was measured at least three times. Isolated exosomes from ES products of AC L4 were placed on a formvar-coated copper grid and settled for 2 min. The grids were washed with deionized water. Then, the sample was contrasted by adding an aqueous solution of 1% phosphotungstic acid for 2 min, followed by a rinse with deionized water. The grid was visualized using Hitachi H7650 transmission electron microscope (Hitachi-Science & Technology). For exosome uptake assay, BMDM cells were incubated with PBS or with 5 μg/ml PKH26-labeled exosomes for 60 min. Subsequently, cells were fixed with 4% paraformaldehyde, permeabilized with 1% Triton X-100, blocked with 5% BSA, and stained with Alexa Fluor Phalloidin-488 as well as DAPI, followed by a confocal microscope analysis using Nikon C2. Scale bar=20 μm.

### Protein and mRNA Analysis

For SDS-PAGE analysis, 30 μg of protein sample was loaded on the SDS-PAGE gel and run in SDS-PAGE running buffer. Then, the protein gels were stained with the coomassie blue staining or silver staining method, and the image was acquired and evaluated by SmartGelTM (Sagecreation Inc., China). Protein molecular weight standards were obtained from Bio-Rad (Thermo Scientific, USA). Lectin blot analysis was performed as described previously (25). The following reagents from VectorLaboratories were used in this experiment: Lectin Kit III, Biotinylated (Cat. No: BK-3000), VECTASTAIN^®^ Elite^®^ ABC HRP Kit (Peroxidase, Standard) (Cat. No: PK-6100), Carbo-Free Blocking Solution (10×Concentrate). Protein Deglycosylation assay was performed using Affi-Gel Hz Immunoaffinity Kit (Biorad, #1536060) as described previously (24). And PNGase F was used to remove almost all N-linked oligosaccharides from glycoproteins. For mRNA analysis, qRT-PCR was carried out using RevertAid™ FirstStrand cDNA Synthesis Kit (Thermo Fisher Scientific, USA) according to the manufacturer’s protocol. Specific gene expression was quantified with SYBR^®^ Premix Ex Taq™ (Tli RNaseH Plus) (RR420A) using the Roche LightCycler^®^ 480 real-time PCR platform. The following amplification primers (Sangon Biotech) were used (5′ to 3′) (**Supplementary Table 1**).

### RNA-Seq and Proteomics Analysis

Proteomics analysis of exofree (10~20 kDa fractions) and exosomes was performed by Jingjie PTM Biolab (Hangzhou, China). For each category of proteins, InterPro database (a resource that provides functional analysis of protein sequences by classifying them into families and predicting the presence of domains and important sites) was researched, and a two-tailed fisher’s exact test was employed to test the enrichment of the identified protein against all proteins. Protein domains with a corrected p-value < 0.05 were considered significant. Soft motif-x was used to analyze the model of sequences constituted with amino acids in specific positions of modify-21-mers (10 amino acids upstream and downstream of the site) in all protein sequences. And all the database protein sequences were used as background database parameters, other parameters with default. BMDMs were treated with PBS, exofree, IL-4, or exofree+IL-4 for 6, 12, 24h, respectively, and the RNA-seq analysis was performed by Novogene Bioinformatics Technology Cooperation (Beijing, China). To predict potential interactions between worm proteins and mouse genes, we developed a bi-layer network method by integrating the RNA-seq data and the proteomics data. First, a gene-gene network was constructed for the TCGs using LASSO regression model based on the RNA-seq expression data. A dynamic network entropy (DNE) index (26) was calculated for each node to measure the importance of genes in the constructed network by taking into account dynamic gene interactions. Subsequently, a protein-gene network was constructed for worm-proteins with molecular weight less than 50 kDa and TCGs of exofree-, IL-4-, and exofree+IL-4-treated BMDMs by using String database (https://string-db.org/). Finally, a bi-layer network for worm-protein and mouse genes was built by integrating the above two networks. Cytoscape software was used to visualize the constructed networks. GO biological process significantly enriched TCGs in exofree-treated BMDMs were identified using R package clusterprofiler (v.3.8.1).

### Statistical Methods

All analyses were performed using GraphPad Prism 6.0 (GraphPad Software, Inc. USA) or SPSS v22 (IBM, USA) software. Statistical analyses of data were performed using Student’s independent samples t-test. Data are expressed as arithmetic mean ± SD, and statistical significance is defined as P < 0.05 (two-sided).

## Results

### Excretory-Secretory Products of AC L4 Promote M2 Polarization of Macrophages

**Figure 1:**
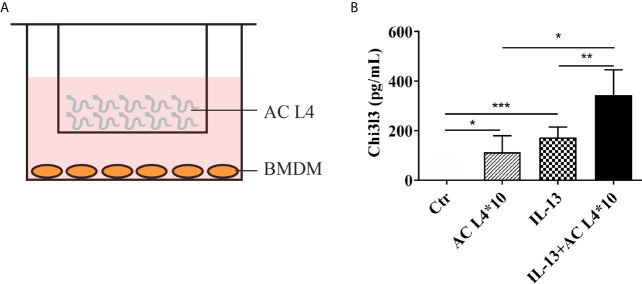
Excretory-secretory products in AC L4 induce M2 polarization of macrophage. **(A)** Diagram of co-culture system with 10 AC L4 above the membrane and BMDMs below the membrane. **(B)** ELISA analysis of Chi3l3 of BMDMs in the presence of 10 AC L4, IL-13, and IL-13+10 AC L4 for 24 h. Data information: *P<0.05, **P<0.01.

A co-culture system of BMDMs and freshly isolated live AC L4 (10 worms per well) was established as shown (**Figure 1A**), and Chi3l3 protein level was measured after 12 h. We observed a significant increase in the protein level of Chi3l3 in BMDMs treated with IL-13 and 10 AC L4. In addition, a striking increase of Chi3l3 level was observed when BMDMs were treated with 10 AC L4 in the presence of IL-13 (**Figure 1B**), suggesting that the ES products of AC L4 could significantly promote macrophage M2 polarization. These results are consistent with our previous finding in BMDMs treated with AC L4 sAg (2, 27).

### Isolation and Characterization of Exosome and Exofree From AC L4 Excretory-Secretory Products

**Figure 2:**
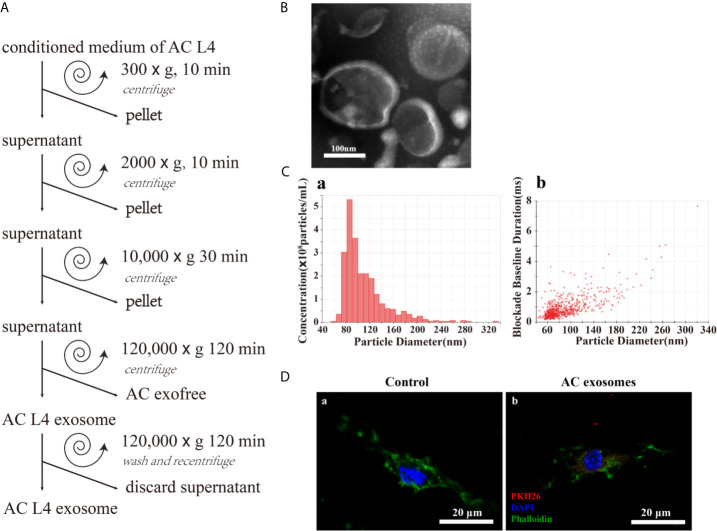
Excretory-secretory products from the fourth stage larva of *A. cantonensis* could be further classified into AC L4 exosome and AC L4 exofree. **(A)** Scheme of AC L4 exosomes and AC L4 exofree isolation by differential ultracentrifugation from conditioned medium of the fourth stage larva of *A. cantonensis*. **(B)** Morphological characterization of AC L4 exosomes were identified using transmission electron microscope. **(C)** Concentration and size distribution of AC L4 exosomes were measured by IZON qNano particle analyzer. **(D)** BMDMs were incubated with PBS **(a)** or 5 μg/ml PKH26-labeled AC L4 exosomes **(b)** for 1 h, and a confocal analysis was performed to evaluate exosome internalization. Phalloidin was used to label actin (green), and DAPI was used for the staining of nuclei (blue). Scale bar=20 μm.

In order to determine the key components in inducing the alternative activation of macrophages, exosomes and exofree were isolated from the ES product of AC L4 using modified differential centrifugation (**Figure 2A**). Transmission electron microscopy (TEM) (**Figure 2B**) and IZON qNano particle analyzer (**Figure 2C**) were used to examine the size and morphology of AC L4 exosome. The electron micrographs of the exosomes revealed rounded structures with a size from 60 to 200 nm, as previously shown (23), consistent with the results of IZON qNano particle analyzer.

It has been proven that helminth-derived microRNAs or proteins-containing exosomes could be internalized by immune cells, exerting an expansion of inflammatory response (7, 28). Likewise, AC L4 exosome could be internalized by BMDMs, confirmed by confocal microscopy (**Figure 2D**). Interestingly, the silver staining results (**Supplementary Figure 1A**) showed that the protein content of AC L4 exosome, AC L4 exofree, and sAg (2, 27) varies from each other.

### AC L4 Exofree, Not AC L4 Exosome, Plays the Main Role in Promoting M2 Polarization of Macrophages

**Figure 3:**
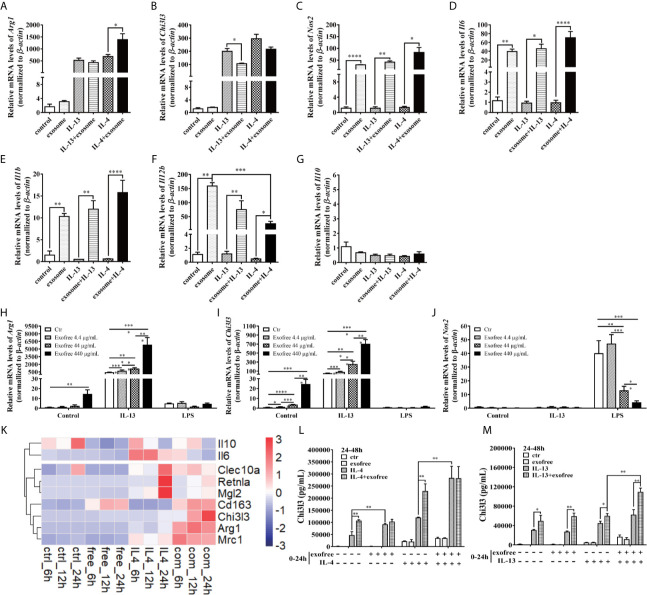
AC L4 exofree could promote a M2 polarization, while AC L4 exosomes induce M1 polarization of macrophage. **(A–G)** Macrophage activation markers *Arg1* **(A)**, *Chi3l3* **(B)**, *Nos2* **(C)**, and related interleukins *Il6* **(D)**, *Il1b* **(E)**, *Il12b* **(F),** and *Il10* **(G)** expression level were measured in the presence of AC L4 exosome, IL-4, IL-4+exosome, IL-13, and IL-13+exosome for 24 h using qPCR. The working concentration of AC L4 exosome is 25 μg/ml. The working concentration of IL-13 and IL-4 is 10 ng/ml. **(H–J)** Macrophage activation markers *Arg1* **(H)**, *Chi3l3* **(I)**, *Nos2* **(J)** were measured in the presence of exofree, IL-4, IL-4+exofree, IL-13, and IL-13+exofree for 24 h using qPCR. **(K)** Signature gene expression profile of macrophage in the presence of PBS (ctr), free (AC L4 exofree), IL-4, com (IL-4+AC L4 exofree), and 6, 12, and 24 h represent 6, 12, and 24 h after the treatment, respectively. The working concentration of AC L4 exofree is 440 μg/ml. The working concentration of IL-13/IL-4 is 10 ng/ml. The working concentration of LPS is 0.05 μg/ml. The horizontal axis represents genes, and vertical coordinates represent types of treatment. **(L, M)** BMDMs were stimulated with PBS, AC L4 exofree, IL-4, IL-4+ AC L4 exofree, IL-13, IL-13+ AC L4 exofree in 0~24 h The culture medium was discarded. BMDMs were then washed with PBS and restimulated in 24~48 h as shown. And Chi3l3 protein level of 24~48 h culture medium was measured using ELISA. The working concentration of AC L4 exofree is 440 μg/ml. The working concentration of IL-13/IL-4 is 10 ng/ml. The detailed experiment information refer to **Supplementary Table 2**. Data information: *P<0.05, **P<0.01, ***P<0.001, ****P<0.0001.

AC L4 exosome and AC L4 exofree were further studied for their role in macrophage activation. AC L4 exosome slightly upregulates Arg1 (**Figure 3A**) mRNA level in BMDMs initiated by IL-4 but does not significantly influence Arg1 and Chi3l3 mRNA level in BMDMs stimulated with AC L4 exosome alone (**Figure 3B**), implying that AC L4 exosome might not significantly affect M2 polarization of macrophages. An increase in inflammatory cytokines, such as *Nos2* (**Figure 3C**), *Il6* (**Figure 3D**), *Il1b* (**Figure 3E**), and *Il12b* (**Figure 3F**), was also observed in BMDMs treated with AC L4 exosome, even in the presence of IL-4 or IL-13, suggesting that it could induce a remarkable M1 polarization of macrophages. However, *Il10* mRNA level (**Figure 3G**) was undetectable in BMDMs, suggesting M1 polarization of macrophage induced by AC L4 exosome is different from that induced by LPS (29). Intriguingly, AC L4 exofree elicits a dose-dependent *Arg1* (**Figure 3H**) and *Chi3l3* (**Figure 3I**), M2-specific genes initiated by IL-13, and a dose-dependent suppression of *Nos2* (**Figure 3J**), an M1-specific gene induced by LPS in macrophages. RNA-seq results also suggested that macrophage M2 signature genes, including *Cd163*, *Chil3*, *Arg1*, *Nos2*, *Clec10a*, *Retnla*, *Pdcd1lg2*, are sharply increased in IL-4, IL-4, and AC L4 exofree co-treated group (**Figure 3K**) at 6, 12, and 24 h. However, cytokines, chemokines, and secreted mediators associated with macrophage polarization were not significantly changed (**Supplementary Figure 2**). Still, a low level of IL-10 in all the groups suggests that M2 polarization of macrophage induced by AC L4 exofree was not caused by hemoglobin or IL-10 (30, 31).

To further determine the role of AC L4 exofree in inducing macrophage polarization, BMDMs were pretreated in 0~24 h. BMDM culture medium was discarded. BMDMs were washed with PBS for three times, followed by indicated treatments in 24~48 h. Finally, the 24~48 h-culture medium was collected, and the Chi3l3 protein level was measured by ELISA (**Figures 3L, M**). For the detailed experiment information, refer to **Supplementary Table 2**. When compared with “PBS”-treatment (0~24 h) BMDMs, IL-4 treatment (24~48 h) could induce a significant upregulation of Chi3l3 level in “exofree”-treated (0~24 h) BMDMs (**Figure 3L**). Intriguingly, in “exofree” pretreated (0~24 h) BMDMs, “IL-4+exofree” treatment (24~48 h) produced a comparable amount of Chi3l3 as “IL-4” treatment (24~48 h) (**Figure 3L**). Similarly, IL-4 treatment (24~48 h) could induce a significant upregulation of Chi3l3 level when BMDMs were treated with “IL-4+exofree”-treated (0~24 h) (**Figure 3L**), compared with “IL-4”-treatment (0~24 h) BMDMs. Intriguingly, in “IL-4+exofree” pretreated (0~24 h) BMDMs, “IL-4+exofree” treatment (24~48 h) produced a comparable amount of Chi3l3 as “IL-4” treatment (24~48 h) (**Figure 3L**). However, these phenotypes above were not observed in BMDMs stimulated with IL-13 (**Figure 3M**). Collectively, these results indicated that AC L4 exofree might act as a switch in promoting M2 polarization of macrophages. And it could be of great value to explore the key molecules in AC L4 exofree.

### Metabolic Reprogramming by the PI3K-Akt Pathway Plays a Vital Role in AC L4 Exofree–Induced M2 Polarization of Macrophages

**Figure 4:**
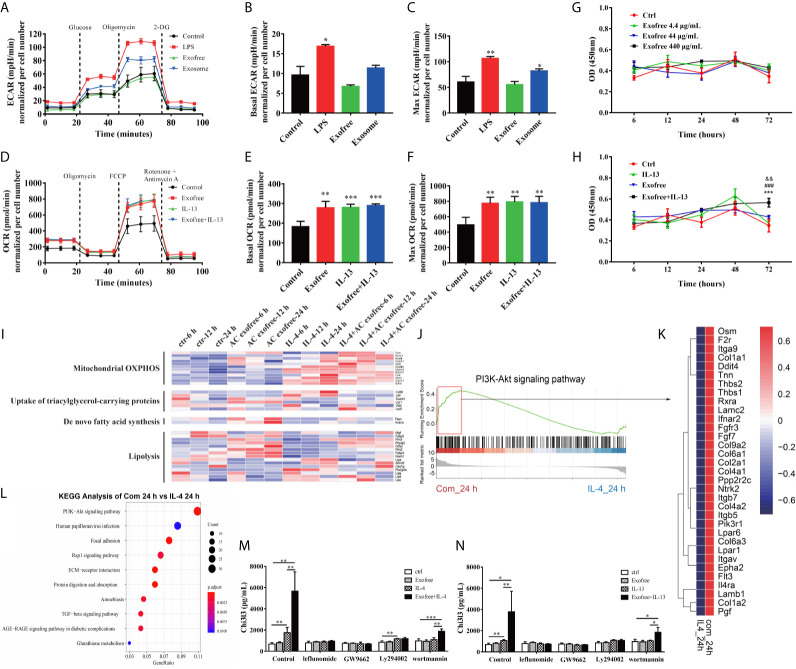
Metabolic Reprogramming by the PI3K-Akt pathway plays a vital role in AC L4 exofree–induced M2 polarization of macrophages. **(A–C)** BMDMs were cultured for 24 h in medium alone or treated with LPS, exofree, exosome, and then the ECAR **(A)** was monitored using the Seahorse Bioscience extracellular flux analyzer in real time. Dotted lines indicate incubation of cells with the indicated compounds. Basal ECAR **(B)** and Max ECAR **(C)** of BMDMs were calculated. *P<0.05, **P<0.01, compared with the control group. In this experiment, LPS concentration is 0.05 μg/ml, AC L4 exosome concentration is 25 μg/ml, and AC L4 exofree concentration is 44 μg/ml. **(D–F)** BMDMs were cultured for 24 h in medium alone or treated with exofree, IL-13, or exofree+IL-13, and then the OCR **(D)** was monitored using the Seahorse Bioscience extracellular flux analyzer in real time. Dotted lines indicate incubation of cells with the indicated compounds. Basal OCR **(E)** and Max ECAR **(F)** of BMDMs were calculated. **P<0.01, ***P<0.001, compared with the control group. In this experiment, IL-13 concentration is 10 ng/ml, and AC L4 exofree concentration is 44 μg/ml. **(G, H)** Proliferation of BMDMs was determined with CCK8 assay at 6, 12, 24, 48, 72 h after treatment of PBS and exofree **(G)**. AC L4 exofree concentration in this experiment is 4.4, 44, 440 μg/ml. **(H)** Proliferation of BMDMs was determined with CCK8 assay at 6, 12, 24, 48, 72 h after treatment of PBS, IL-13, exofree, IL-13+exofree. In this experiment, AC L4 exofree concentration is 440 μg/ml, and IL-13 concentration is 10 ng/ml. *^&&^P*<0.01, compared with the control group; *^###^P*<0.001, compared with the IL-13 group; ***P<0.001, compared with the exofree group. **(I)** Gene expression profile of BMDMs related to mitochondrial OXPHOS, nutrient uptake, *de novo* fatty acid synthesis, and lipolysis metabolism process were shown in a heatmap. BMDMs were treated with PBS (ctr), free (AC L4 exofree), IL-4, com (IL-4+AC L4 exofree) for 6, 12, and 24 h, respectively. The horizontal axis represents genes, and vertical coordinates represent types of treatment. In this experiment, AC L4 exofree concentration is 440 μg/ml, and IL-4 concentration is 10 ng/ml. **(J)** GSEA analysis of genes enriched in PI3K-Akt signaling pathway was performed using R package “clusterprofiler.” **(K)** Signature genes enriched in PI3K-Akt signaling pathway through GSEA analysis were shown in a heatmap. **(L)** GO enrichment analysis (biological process) of highly expressed genes in the presence of IL-4 and AC L4 exofree, compared with IL-4 treatment, was performed using R package “clusterprofiler.” **(M, N)** BMDMs were treated with PBS, exofree, IL-13, IL-13+exofree **(M)**, and PBS, exofree, IL-4, IL-4+exofree **(N)** in the presence of leflunomide (a STAT6 inhibitor), GW9662 (a PPARγ antagonist), Ly294002 (PI3K inhibitor), and wortmannin (PI3K inhibitor) for 24 h. The protein levels of Chi3l3 in the culture medium were measured using ELISA. The following reagents were used in this experiment: Leflunomide (100 μM), Ly294002 (10 μM), Wortmannin (100 nM), GW9662 (1 μg/ml). *P<0.05, **P<0.01.

To functionally validate whether metabolism is affected in BMDMs treated with AC L4 exosomes or AC L4 exofree, BMDMs were treated with AC L4 exosome and AC L4 exofree for 24 h, and the oxygen consumption rate (OCR) and extracellular acidification rate (ECAR) were measured. Consistent with the level of Arg1 (**Figure 3H**) and Chi3l3 (**Figure 3I**), elevated basal OCR and max OCR indicated an enhanced oxidative phosphorylation metabolic phenotype in BMDMs when treated with IL-13, AC L4 exofree, or a combination of IL-13 and AC L4 exofree (**Figures 4D–F**). However, neither basal nor max ECAR (**Supplementary Figure 3C**) level of BMDMs changed if treated with AC L4 exofree alone. Meanwhile, both OCR (**Figures 4D–F**) and ECAR levels (**Figures 4A–C**) were markedly elevated in BMDMs treated with AC L4 exosome.

Consistent with the OCR results of exofree-treated BMDMs, relative transcript levels of genes related to mitochondrial oxidative phosphorylation (OXPHOS) (**Figure 4I**), β-oxidation (**Supplementary Figure 3A**), lipolysis (**Figure 4I**) also changed accordingly, while nutrient uptake and *de novo* fatty acid synthesis were not significantly affected (**Figure 4I**) in macrophages treated with AC L4 exofree+IL-4/IL-13. Importantly, polarization and metabolic reprogramming of macrophages might be partially associated with BMDMs proliferation (**Figures 4G, H**) (**Supplementary Figure 3D**). However, M2 polarization of macrophage was severely affected whether glycolysis or OXPHOS was inhibited (**Supplementary Figures 3E, F**).

To define the key signal pathway, GO analysis was performed based on the RNA-seq results of BMDMs treated with AC L4 exofree+IL-4 and IL-4 for 24 h. And PI3K-Akt pathway was identified as the top enriched signaling pathway (**Figure 4L**). The upregulated genes are as follows (**Figures 4J, K**).

The results showed that when BMDMs were treated with leflunomide, GW9662, PI3K-specific inhibitor (Ly294002 and wortmannin), Chi3l3 level in IL-4/IL-13-treated BMDMs showed no difference from Chi3l3 level in PBS-treated BMDMs, exofree-treated BMDMs, and “IL-4/IL-13+exofree”-treated BMDMs, suggesting JAK-STAT pathway and PPARγ pathway are sufficient for M2 polarization of macrophages. Interestingly, in wortmannin-treated BMDMs, Chi3l3 level in “exofree+IL-4,” “exofree+IL-13” group is a little bit higher than that in “IL-4,” “IL-13” group respectively, suggesting that exofree-induced Chi3l3 expression might be partially associated with PI3K signaling (**Figures 4M, N**). These results demonstrate that AC L4 exosomes and exofree could differently affect macrophage activation through metabolic reprogramming. And PI3K-Akt pathway might play a vital role in AC L4 exofree–induced M2 polarization of macrophages.

### Glycosylated Analogs From AC L4 Exofree Could Be the Key Ingredient in Enhancing Alternative Activation of Macrophage

**Figure 5:**
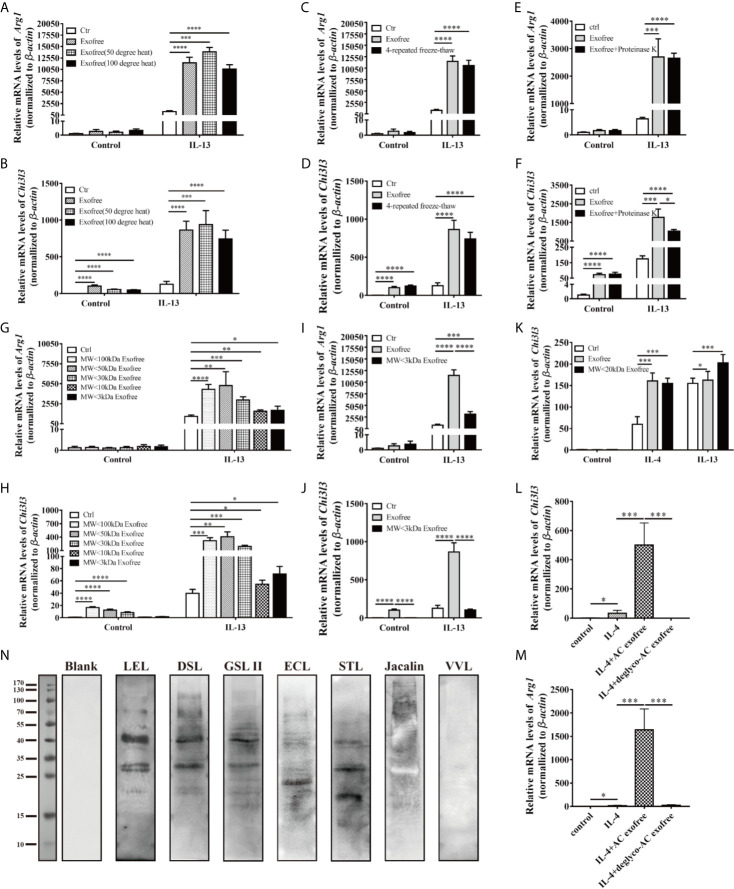
Chemical and physical property analysis of AC L4 exofree suggested glycoproteins as the key components in AC L4 exofree–induced M2 polarization of macrophage. **(A, B)** qPCR analysis of *Arg1* **(A),** *Chi3l3* **(B)** mRNA level of BMDMs in the presence of PBS, AC L4 exofree, AC L4 exofree (50°C water bath, 15min), AC L4 exofree (100°C water bath, 15min), IL-13, AC L4 exofree+IL-13, AC L4 exofree (50°C water bath, 15min)+IL-13, AC L4 exofree (100°C water bath, 15 min)+IL-13 at 24 h was performed. **(C, D)** qPCR analysis of Arg1 **(C)**, Chi3l3 **(D)** mRNA level of BMDMs in the presence of PBS, AC L4 exofree, AC L4 exofree (repeated freeze-thaw cycles for four times) at 24 h was performed. **(E, F)** qPCR analysis of Arg1 **(E)**, Chi3l3 **(F)** mRNA level of BMDMs in the presence of PBS, AC L4 exofree, AC L4 exofree (100 μg/ml proteinase K treatment: 58°C water bath, 2 h; proteinase K inactivation: 100°C water bath, 15 min) at 24 h was performed. **(G, H)** qPCR analysis of Arg1 **(G)**, Chi3l3 **(H)** mRNA level of BMDMs in the presence of PBS, AC L4 exofree, AC L4 exofree (<3 kDa fraction), AC L4 exofree (<10 kDa fraction), AC L4 exofree (<30 kDa fraction), AC L4 exofree (<50 kDa fraction), AC L4 exofree (<100 kDa fraction), IL-13, AC L4 exofree+IL-13, AC L4 exofree (<3 kDa fraction)+IL-13, AC L4 exofree (<10 kDa fraction)+IL-13, AC L4 exofree (<30 kDa fraction)+IL-13, AC L4 exofree (<50 kDa fraction)+IL-13, AC L4 exofree (<100 kDa fraction)+IL-13, at 24 h was performed. **(I, J)** qPCR analysis of Arg1 **(I)** and Chi3l3 **(J)** mRNA level of BMDMs in the presence of PBS, AC L4 exofree, AC L4 exofree (<3 kDa fraction), IL-13, AC L4 exofree+IL-13, AC L4 exofree (<3 kDa fraction)+IL-13 at 24 h was performed. In **(A–J)**, the concentration of exofree is 440 μg/ml and the concentration of IL-13 is 10 ng/ml. **(K)** qPCR analysis of Chi3l3 mRNA level of BMDMs in the presence of PBS, AC L4 exofree, AC L4 exofree (<20 kDa fraction), IL-13, AC L4 exofree+IL-13, AC L4 exofree (<20 kDa fraction)+IL-13 at 24 h was performed. In **(K)**, the concentration of exofree is 44 μg/ml, the concentration of IL-13 is 10 ng/ml. **(L, M)** Control (PBS) or AC L4 exofree or deglycosylated AC L4 exofree were used to stimulate BMDMs. And mRNA expression level of Chi3l3 **(L)** and Arg1 **(M)** were measured 24 h after the stimulation using qPCR. **(N)** Glycoproteins in AC L4 exofree were measured by lectin blot. Membranes were incubated with PBS (lane Blank), lectin LEL, DSL, GSL II, ECL, STL, Jacalin, and VVL as primary antibody, respectively. The same amount of exofree is used in experiments from **(A–M)**. Data information: *P<0.05, **P<0.01, ***P<0.001, ****P<0.0001.

Accumulating findings have suggested the predominance of M1 macrophages in autoimmune diseases, such as inflammatory bowel disease and systemic lupus erythematosus (32). Considering macrophages are critical mediators of the immune responses, and AC L4 exofree could significantly inhibit pro-inflammatory M1 macrophages and promote M2 anti-inflammatory macrophages, thus defining the key ingredient might contribute to drug development for immune-related diseases. To identify the key components of AC L4 exofree, it was then fractionated by size (<3, <10, <20, <30, <50, and <100 kDa, respectively) using ultracentrifugal filters, and the activity of all the fractions was tested. Unfractionated AC L4 exofree and the <20 kDa fraction induce a similar mRNA level of *Chi3l3*; however, the <30 kDa fraction failed to effectively upregulate *Arg1* and *Chi3l3* expression, suggesting the M2-related molecules in AC exofree were neither small-molecule metabolites (**Figures 5I, J**) (**Supplementary Figure 4C**) nor macromolecules (**Figures 5G–K**), which have a molecular weight from 10 to 20 kDa (**Figures 5G–K**).

Intriguingly, although exposed under 15 min-50 or 100°C water baths (**Figures 5A, B** and **Supplementary Figure 4A**), four-time freeze-and-thaw (**Figures 5C, D**), or protease K-treatment (**Figures 5E, F** and **Supplementary Figure 4B**), the function of AC L4 exofree remained stable.

We next sought to define the biochemical property of exofree. Protein glycosylation, one of the most important post-translational modifications, has been proven essential in many biological processes, especially enzyme activity, protein stability, and receptor-ligand binding. As N-glycosylation (33), but not O-glycosylation or fucosylation, in AC has been proven essential for immune recognition (34), we speculate N-linked glycoprotein in AC L4 exofree might play a role in promoting M2 polarization of macrophages. As expected, lectin blot (**Figure 5N**) analysis showed that AC L4 exofree contains N-linked glycoproteins. Deglycosylation experiment suggested that AC L4 exofree without N-linked glycosylation failed to facilitate M2 polarization of macrophage (**Figures 5L, M**), suggesting glycosylated analogs from AC L4 exofree could be the key ingredient in enhancing alternative activation of macrophages.

### Bi-layer PPI Network Analysis Suggests an AC L4 Exofree-Macrophage Interaction Through Hexa

**Figure 6:**
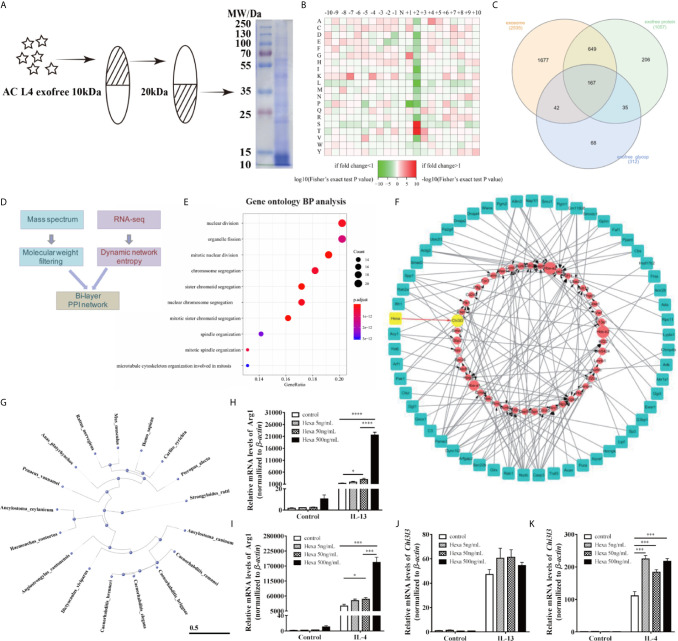
AC-derived Hexa act as a key ingredient in promoting M2 activation of macrophages. **(A)** Diagram of isolation of 10~20 kDa-AC L4 exofree using ultrafiltration tubes, and the efficiency was confirmed using western blot. **(B)** Heatmap showed the relative frequencies of amino acids between the asparagine (Asn) position of N-linked glycoproteins in AC L4 exofree. Enrichment level of the amino acid at indicate position is shown in different colors. Red means high enrichment level, and green means low enrichment level. **(C)** Venn diagram of AC L4 exosome, AC L4 exofree proteins identified using mass spectrum analysis. **(D)** Diagram of Bi-layer PPI network analysis using AC L4 exofree proteins and RNA-seq data of BMDMs. **(E)** GO enrichment analysis (Biological process) of exofree-induced TCGs of BMDMs (**Supplementary Figure 5B**). **(F)** A protein-gene network was constructed for worm proteins with molecular weight less than 50kDa and TCGs of exofree-, IL-4-, and exofree+IL-4-treated BMDMs (**Supplementary Figure 5A**), based on the LC-MS/MS and RNA-seq data. **(G)** Phylogene tree of Hexa protein in 17 species. **(H, J)** qPCR analysis of mRNA level of Arg1 **(H)** and Chi3l3 **(J)** were measured in the presence of PBS, Hexa 5 ng/ml, Hexa 50 ng/ml, Hexa 500 ng/ml, IL-13 10 ng/ml, IL-13+Hexa 5 ng/ml, IL-13+Hexa 50 ng/ml, IL-13+Hexa 500 ng/ml for 24 h. **(I, K)** qPCR analysis of mRNA level of Arg1 **(I)** and Chi3l3 **(K)** were measured in the presence of PBS, Hexa 5 ng/ml, Hexa 50 ng/ml, Hexa 500 ng/ml, IL-4 10 ng/ml, IL-4+Hexa 5 ng/ml, IL-4+Hexa 50 ng/ml, IL-4+Hexa 500 ng/ml for 24 h. Data information: *P<0.05, ***P<0.001, ****P<0.0001.

Based on our prediction of the molecular weight of M2 macrophage-related molecules in AC L4 exofree, its 10~20 kDa ingredient was collected using ultrafiltration tubes as shown, and the efficiency was further confirmed using SDS-PAGE followed by coomassie blue staining (**Figure 6A**). Protein mass spectrum analysis of AC L4 exofree (10~20 kDa) was performed, and 312 N-linked glycoproteins were identified (**Figures 6B, C**). Amino acid composition heatmap (**Figure 6B**) suggested that serine (S) and threonine (T) were significantly enriched at position +2 N-glycosylation site, indicating that N glycoproteins derived from AC L4 exofree are conserved, with a consensus N-glycosylation “sequon” (AsnXxxSer/Thr/Cys, where Xxx can be any amino acid except proline) (35). Compared with protein mass spectrometry analysis of AC L4 exosome, 35 potential M2-related protein was selected.

RNA-seq analysis of BMDMs with different treatments at different time points was performed (**Supplementary Figure 5A**). Significant temporally changing genes (TCGs) on BMDMs were selected accordingly (2), which could be classified into six groups based on their expression profiles (**Supplementary Figure 5B**). GO biological process enrichment analysis of exofree-induced TCGs of BMDMs (**Figure 6E**) showed that the genes with the time-increasing pattern were mainly enriched in nuclear division. By integrating RNA-seq expression data and proteomics data, we developed a bi-layer network approach (**Figure 6D**) to predict important genes and potential interactions between AC proteins and mouse genes. The bi-layer network (**Figure 6F**) prioritized Chi3l3, a marker of M2 type macrophage, as an important gene and suggested that Hexa protein in AC L4 exofree is functionally related to M2 macrophage marker Chi3l3. Phylogenetic tree of Hexa protein in 17 species including *Homo sapiens* and *M. musculus* was drawn using a constraint-based alignment tool (**Figure 6G**). Amino acid sequence alignment of AC and human Hexa protein was performed using ESPript 3 (**Supplementary Figure 6**). To directly test whether Hexa was the main driver of this effect, BMDMs were treated with recombinant human Hexa, as the high sequence consistency with AC Hexa, and the results showed a dose-dependent increase of Arg1, but not Chi3l3 expression (**Figures 6H–K**) in BMDMs. These results demonstrate that AC L4 exofree might promote alternative activation of macrophage through Hexa.

## Discussion

Macrophages are key participants in AC-host interaction. Previous studies have shown that AC L4-derived soluble antigen, without any IL-4, IL-13, IL-33, and IL-6 analogs (unpublished data), could promote M2 polarization of macrophage in a PPARγ-dependent manner (27). However, the important compound or protein in AC-derived soluble antigen is not clear.

During the recent few years, exosomes and other extracellular vesicles have been isolated and characterized in all known pathogen classes, including parasites. The research on parasite-derived exosomes has expanded dramatically. Parasite-derived exosomes are capable of modulating immune responses through regulating the functions of macrophages. Extracellular vesicles of *Heligmosomoides polygyrus* could suppress both M1 and M2 polarization of macrophages (36). Interestingly, M2 polarization of macrophages and worm burden could be significantly inhibited using antibody-mediated EV uptake block (36). *Leishmania major* exosomes were reported to play a role in the establishment of parasite infection (37). Injection of *Trypanosoma cruzi* extracellular vesicles resulted in a significant reduction of iNOS production and an increase of parasitism in internal organs in *Trypanosoma cruzi*–infected mice (38). However, in some studies, exosomes play a role in generating immunity against parasitic infections. *Toxoplasma gondii*–derived exosomes were reported to induce protective immunity by upregulating IFN-γ, TNF-α, and IL-12 in macrophages (39). Adult *Schistosoma japonicum*–derived exosome-like vesicles were reported to induce macrophage polarization to an M1 phenotype (40). Confusingly, M1 macrophages are favorable to kill schistosomula by producing NO (41, 42), preventing hepatic fibrosis.

Differential centrifugation is a recommended method to separate exosomes and exosome-depleted ES products. Exosomes are small membrane-bound vesicles and have proven to be stable, which could mediate long-range communication with distant targets (43).

It has been reported that proteins in exosome-like vesicles of *Schistosoma japonicum* are significantly different from soluble worm antigenic preparations, and debris, extracellular vesicle (EV) from ES products (40). Similarly, EV content is significantly different from that in EV-depleted ES products and ES of *H. polygyrus* (36). However, rare studies are focused on the exosome-depleted component, which also might play a more vital role than exosomes in tissue hemostasis.

In this study, exosomes and exosome-depleted ES products, exofree, were obtained, and their distinct roles in regulating macrophage polarization were firstly confirmed. AC L4 exosome could induce a significant M1, not M2, polarization of macrophages. This might partially clarify the reason why microglia (44), brain-resident macrophages, are classically activated in AC infection. However, AC L4 exofree, heat and protease-stable, could significantly promote M2 polarization induced by IL-4/IL-13, while inhibiting M1 polarization of macrophage induced by LPS. These results strongly cue that when studying the function of parasitic exosomes, we should also pay attention to other components within excretory/secretory products.

Lectin blot and deglycosylation assay reinforce the important role of glycosylated products of AC L4 exofree on macrophage polarization (**Figure 5M**). Therefore, N-glycoproteins in AC L4 exofree were identified using mass spectrometry–based glycoproteomics (24). Then, a PPI network analysis was performed based on AC L4 exofree-derived N-glycoproteins, and BMDMs differentially expressed genes regulated by AC L4 exofree, and Hexa was predicted to be the key molecule in M2 macrophage polarization. Hexosaminidase, a lysosomal enzyme involved in the breakdown of gangliosides, consists of α and/or β subunit, which is encoded by Hexa gene and Hexb gene, respectively, forming three isozymes—hexosaminidase A (α/β heterodimer), hexosaminidase B (β/β homodimer), and hexosaminidase S (α/α homodimer). Hexb, a peptidoglycan hydrolase, could either kill mycobacteria directly or restrict its growth in an IFNγ-dependent manner (45). Functional assay of AC Hexa was performed using human Hexa protein, an analog recombinant protein produced by *E. coli*, suggesting a role of Hexa in promoting M2 polarization of macrophage induced by IL-4/IL-13. However, Arg1, but not Chi3l3, in BMDMs could be upregulated in a Hexa dose-dependent manner.

Intriguingly, M2 macrophage polarization induced by Hexa seems unrelated to its enzymic activity, as Hexa protein displayed a single-phase inactivation curve at 48°C (46), and Hexb protein behaves as heat-stable enzyme activities at 55°C (47). A comparative functional study of Hexa and Hexb in macrophage polarization could be carried out in the future. However, the coding sequences of AC Hexa/Hexb still remain to be identified. As proteins produced from *E. coli* are lacking of sugar chain, which is of great importance for protein antigenicity (33) and stability (48), eukaryotic protein expression systems could be used in our study. Previous studies have shown that N-glycans, but not O-glycans or fucose-containing glycans, from AC L4 seem essential for immune recognition (34); thus, α-L-Fucosidase, Endo H, and O-glycosidase might help exclude the possible role of O-glycosylation or fucosylation in this process.

Energy metabolism has been proven to be an mTOR-dependent cellular process, and inhibition of mTOR could induce a switch of energy metabolism from mitochondrial oxidative phosphorylation to aerobic glycolysis (46). Inhibition of mTORC1 could significantly upregulate M1-associated cytokines level (49), and both mTORC1 (50) and mTORC2 (51) seem critical for M2 macrophages. Based on our previous study on macrophage and KEGG results of BMDMs stimulated with AC L4 exofree, we supposed PI3K/Akt, an upstream modulator of the mTORC1 signal pathway, might play an important role in macrophage M2 polarization induced by AC L4 exofree. Expectedly, class I PI3K inhibitor Ly294002 and wortmannin successfully induced a sharp decrease of Chi3l3 protein level in BMDMs treated with “AC L4 exofree+IL-4” and “IL-4.” However, Chi3l3 protein level in BMDMs treated with “AC L4 exofree+IL-4” was significantly higher than IL-4-treated group in the presence of 100 nM wortmannin, but not in 10 μM Ly294002. These results suggested that PI3K/Akt pathway might be partially associated with AC L4 exofree–induced M2 polarization of BMDMs. This might be because of their different inhibitory effects on class II PI3K, and 10 μM LY294002 has a partial inhibitory effect on PI3K-C2β (52), while 100 nM wortmannin is not sufficient to inhibit PI3K-C2α activity (53). Therefore, a deeper understanding of the signaling pathways in this process is needed.

In this study, we present evidence in favor of a key role for N-linked glycoprotein in AC L4 exofree, a component free of exosomes isolated from ES products of AC L4, in promoting M2 macrophage polarization. And metabolic reprogramming by the PI3K-Akt pathway might play an important role in this process. Hexa, a macrophage-related N-linked glycoprotein, might play a key role in directing host CNS inflammation in AC infection. The results of this study indicate a great value in studying ES products of AC L4, as worm-derived immunoregulatory molecules might contribute to drug development for immune-related diseases.

## Data Availability Statement

The datasets presented in this study can be found in online repositories, further inquiries can be directed to the corresponding author. Proteomics data of AC L4 exosomes and AC L4 exofree have been deposited to the ProteomeXchange Consortium via the PRIDE (54) partner repository with the dataset identifier PXD025218. RNA-seq data of BMDMs were deposited in the Gene Expression Omnibus (GEO) datasets under reference number GSE175497. The code used for network analysis in this study is available at https://github.com/SunXQlab/Hexa-network.

## Ethics Statement

The animal study was reviewed and approved by the medical research ethics committee of Sun Yat-sen University (SYSU-IACUC-2019-B535).

## Author Contributions

XS, ZW, and SW conceived the concept, contributed to experimental designs. SW, WT, and LW performed the study and the experiments. XQS conducted RNA-seq and proteomics analysis. SW and XS wrote the manuscript. All authors contributed to the article and approved the submitted version.

## Funding

This project was supported by the Science and Technology Planning Project of Guangdong Province (2016A020219004), the National Key R&D Program of China (2020YFC1200100), Natural Science Foundation of Guangdong Province (No 2019A1515012068, 2021A1515010976), the Pearl River Nova Program of Guangzhou (No. 201710010030), 111 Project (Grant No. B12003). Shuo Wan was supported by the Project funded by China Postdoctoral Science Foundation (2021M691236). Xiaoqiang Sun was supported by the National Natural Science Foundation of China (11871070), the Guangdong Basic and Applied Basic Research Foundation (2020B151502120), the Fundamental Research Funds for the Central Universities (20ykzd20), and Guangdong Key Field R&D Plan (2019B020228001).

## Conflict of Interest

The authors declare that the research was conducted in the absence of any commercial or financial relationships that could be construed as a potential conflict of interest.

## Publisher’s Note

All claims expressed in this article are solely those of the authors and do not necessarily represent those of their affiliated organizations, or those of the publisher, the editors and the reviewers. Any product that may be evaluated in this article, or claim that may be made by its manufacturer, is not guaranteed or endorsed by the publisher.

## References

[1] Zhao J Lv Z Wang F Wei J Zhang Q Li S. Ym1, an Eosinophilic Chemotactic Factor, Participates in the Brain Inflammation Induced by Angiostrongylus Cantonensis in Mice. Parasitol Res (2013) 112:2689–95. 10.1007/s00436-013-3436-x 23703548

[2] Wan S Sun X Wu F Yu Z Wang L Lin D. Chi3l3: A Potential Key Orchestrator of Eosinophil Recruitment in Meningitis Induced by Angiostrongylus Cantonensis. J Neuroinflamm (2018) 15:31. 10.1186/s12974-018-1071-2 PMC5796390 29391024

[3] Rodriguez E Noya V Cervi L Chiribao ML Brossard N Chiale C. Glycans From Fasciola Hepatica Modulate the Host Immune Response and TLR-Induced Maturation of Dendritic Cells. PloS Negl Trop Dis (2015) 9:e0004234. 10.1371/journal.pntd.0004234 26720149 PMC4697847

[4] Prasanphanich NS Luyai AE Song X Heimburg-Molinaro J Mandalasi M Mickum M. Immunization With Recombinantly Expressed Glycan Antigens From Schistosoma Mansoni Induces Glycan-Specific Antibodies Against the Parasite. Glycobiology (2014) 24:619–37. 10.1093/glycob/cwu027 PMC4038251 24727440

[5] Eichenberger RM Talukder MH Field MA Wangchuk P Giacomin P Loukas A. Characterization of Trichuris Muris Secreted Proteins and Extracellular Vesicles Provides New Insights Into Host-Parasite Communication. J Extracell Vesicles (2018) 7:1428004. 10.1080/20013078.2018.1428004 29410780 PMC5795766

[6] Buck AH Coakley G Simbari F McSorley HJ Quintana JF Le Bihan T. Exosomes Secreted by Nematode Parasites Transfer Small RNAs to Mammalian Cells and Modulate Innate Immunity. Nat Commun (2014) 5:5488. 10.1038/ncomms6488 25421927 PMC4263141

[7] Soulat D Bogdan C. Function of Macrophage and Parasite Phosphatases in Leishmaniasis. Front Immunol (2017) 8:1838. 10.3389/fimmu.2017.01838 29312331 PMC5743797

[8] Ramos-Benitez MJ Ruiz-Jimenez C Aguayo V Espino AM. Recombinant Fasciola Hepatica Fatty Acid Binding Protein Suppresses Toll-Like Receptor Stimulation in Response to Multiple Bacterial Ligands. Sci Rep (2017) 7:5455. 10.1038/s41598-017-05735-w 28710478 PMC5511235

[9] Danilowicz-Luebert E Steinfelder S Kuhl AA Drozdenko G Lucius R Worm M. A Nematode Immunomodulator Suppresses Grass Pollen-Specific Allergic Responses by Controlling Excessive Th2 Inflammation. Int J Parasitol (2013) 43:201–10. 10.1016/j.ijpara.2012.10.014 23174104

[10] Ziegler T Rausch S Steinfelder S Klotz C Hepworth MR Kuhl AA. A Novel Regulatory Macrophage Induced by a Helminth Molecule Instructs IL-10 in CD4+ T Cells and Protects Against Mucosal Inflammation. J Immunol (2015) 194:1555–64. 10.4049/jimmunol.1401217 25589067

[11] Prieto-Lafuente L Gregory WF Allen JE Maizels RM. MIF Homologues From a Filarial Nematode Parasite Synergize With IL-4 to Induce Alternative Activation of Host Macrophages. J Leukoc Biol (2009) 85:844–54. 10.1189/jlb.0808459 PMC2691607 19179453

[12] Du L Wei H Li L Shan H Yu Y Wang Y. Regulation of Recombinant Trichinella Spiralis 53-kDa Protein (Rtsp53) on Alternatively Activated Macrophages *via* STAT6 But Not IL-4ralpha *In Vitro* . Cell Immunol (2014) 288:1–7. 10.1016/j.cellimm.2014.01.010 24534206

[13] Robinson MW Donnelly S Hutchinson AT To J Taylor NL Norton RS. A Family of Helminth Molecules That Modulate Innate Cell Responses *via* Molecular Mimicry of Host Antimicrobial Peptides. PloS Pathog (2011) 7:e1002042. 10.1371/journal.ppat.1002042 21589904 PMC3093369

[14] Liu J Zhu L Wang J Qiu L Chen Y Davis RE. Schistosoma Japonicum Extracellular Vesicle miRNA Cargo Regulates Host Macrophage Functions Facilitating Parasitism. PloS Pathog (2019) 15:e1007817. 10.1371/journal.ppat.1007817 31163079 PMC6548406

[15] Nyame AK Lewis FA Doughty BL Correa-Oliveira R Cummings RD. Immunity to Schistosomiasis: Glycans are Potential Antigenic Targets for Immune Intervention. Exp Parasitol (2003) 104:1–13. 10.1016/S0014-4894(03)00110-3 12932753

[16] Khoo KH Dell A. Glycoconjugates From Parasitic Helminths: Structure Diversity and Immunobiological Implications. Adv Exp Med Biol (2001) 491:185–205. 10.1007/978-1-4615-1267-7_14 14533799

[17] Restrepo BI Obregon-Henao A Mesa M Gil DL Ortiz BL Mejia JS. Characterisation of the Carbohydrate Components of Taenia Solium Metacestode Glycoprotein Antigens. Int J Parasitol (2000) 30:689–96. 10.1016/S0020-7519(00)00057-6 10856502

[18] Klaver EJ Kuijk LM Laan LC Kringel H van Vliet SJ Bouma G. Trichuris Suis-Induced Modulation of Human Dendritic Cell Function is Glycan-Mediated. Int J Parasitol (2013) 43:191–200. 10.1016/j.ijpara.2012.10.021 23220043

[19] Everts B Hussaarts L Driessen NN Meevissen MH Schramm G van der Ham AJ. Schistosome-Derived Omega-1 Drives Th2 Polarization by Suppressing Protein Synthesis Following Internalization by the Mannose Receptor. J Exp Med (2012) 209:1753–67. 10.1084/jem.20111381 PMC3457738 22966004

[20] Zheng X Hu X Zhou G Lu Z Qiu W Bao J. Soluble Egg Antigen From Schistosoma Japonicum Modulates the Progression of Chronic Progressive Experimental Autoimmune Encephalomyelitis *via* Th2-Shift Response. J Neuroimmunol (2008) 194:107–14. 10.1016/j.jneuroim.2007.12.001 18207251

[21] Kowal J Arras G Colombo M Jouve M Morath JP Primdal-Bengtson B. Proteomic Comparison Defines Novel Markers to Characterize Heterogeneous Populations of Extracellular Vesicle Subtypes. Proc Natl Acad Sci USA (2016) 113:E968–77. 10.1073/pnas.1521230113 PMC4776515 26858453

[22] Nawaz M Malik MI Hameed M Zhou J. Research Progress on the Composition and Function of Parasite-Derived Exosomes. Acta Trop (2019) 196:30–6. 10.1016/j.actatropica.2019.05.004 31071298

[23] Thery C Amigorena S Raposo G Clayton A. Isolation and Characterization of Exosomes From Cell Culture Supernatants and Biological Fluids. Curr Protoc Cell Biol (2006) 3. 10.1002/0471143030.cb0322s30 18228490

[24] Zhu J Sun Z Cheng K Chen R Ye M Xu B. Comprehensive Mapping of Protein N-Glycosylation in Human Liver by Combining Hydrophilic Interaction Chromatography and Hydrazide Chemistry. J Proteome Res (2014) 13:1713–21. 10.1021/pr401200h 24495048

[25] Sato T. Lectin-Probed Western Blot Analysis. Methods Mol Biol (2014) 1200:93–100. 10.1007/978-1-4939-1292-6_8 25117227

[26] Zhang J Zhu W Wang Q Gu J Huang LF Sun X. Differential Regulatory Network-Based Quantification and Prioritization of Key Genes Underlying Cancer Drug Resistance Based on Time-Course RNA-Seq Data. PloS Comput Biol (2019) 15:e1007435. 10.1371/journal.pcbi.1007435 31682596 PMC6827891

[27] Wu F Wei J Liu Z Zeng X Yu Z Lv Z. Soluble Antigen Derived From IV Larva of Angiostrongylus Cantonensis Promotes Chitinase-Like Protein 3 (Chil3) Expression Induced by Interleukin-13. Parasitol Res (2016) 115:3737–46. 10.1007/s00436-016-5135-x 27256220

[28] Silva VO Maia MM Torrecilhas AC Taniwaki NN Namiyama GM Oliveira KC. Extracellular Vesicles Isolated From Toxoplasma Gondii Induce Host Immune Response. Parasite Immunol (2018) 40:e12571. 10.1111/pim.12571 29974519

[29] Iyer SS Ghaffari AA Cheng G. Lipopolysaccharide-Mediated IL-10 Transcriptional Regulation Requires Sequential Induction of Type I IFNs and IL-27 in Macrophages. J Immunol (2010) 185:6599–607. 10.4049/jimmunol.1002041 PMC4103176 21041726

[30] Etzerodt A Moestrup SK. CD163 and Inflammation: Biological, Diagnostic, and Therapeutic Aspects. Antioxid Redox Signaling (2013) 18:2352–63. 10.1089/ars.2012.4834 PMC3638564 22900885

[31] Philippidis P Mason JC Evans BJ Nadra I Taylor KM Haskard DO. Hemoglobin Scavenger Receptor CD163 Mediates Interleukin-10 Release and Heme Oxygenase-1 Synthesis: Antiinflammatory Monocyte-Macrophage Responses *In Vitro*, in Resolving Skin Blisters *In Vivo*, and After Cardiopulmonary Bypass Surgery. Circ Res (2004) 94:119–26. 10.1161/01.RES.0000109414.78907.F9 14656926

[32] Italiani P Boraschi D. From Monocytes to M1/M2 Macrophages: Phenotypical vs Function Differentiation. Front Immunol (2014) 5:514. 10.3389/fimmu.2014.00514 25368618 PMC4201108

[33] Morassutti AL Levert K Perelygin A da Silva AJ Wilkins P Graeff-Teixeira C. The 31-kDa Antigen of Angiostrongylus Cantonensis Comprises Distinct Antigenic Glycoproteins. Vector Borne Zoonotic Dis (Larchmont NY) (2012) 12:961–8. 10.1089/vbz.2011.0957 PMC3491624 22925026

[34] Veríssimo CM Morassutti AL von Itzstein M Sutov G Hartley-Tassell L McAtamney S. Characterization of the N-Glycans of Female Angiostrongylus Cantonensis Worms. Exp Parasitol (2016) 166:137–43. 10.1016/j.exppara.2016.04.012 27107931

[35] Medzihradszky KF. Characterization of Site-Specific N-Glycosylation. Methods Mol Biol (2008) 446:293–316. 10.1007/978-1-60327-084-7_21 18373266

[36] Coakley G McCaskill JL Borger JG Simbari F Robertson E Millar M. Extracellular Vesicles From a Helminth Parasite Suppress Macrophage Activation and Constitute an Effective Vaccine for Protective Immunity. Cell Rep (2017) 19:1545–57. 10.1016/j.celrep.2017.05.001 PMC5457486 28538175

[37] Hassani K Shio MT Martel C Faubert D Olivier M. Absence of Metalloprotease GP63 Alters the Protein Content of Leishmania Exosomes. PloS One (2014) 9:e95007. 10.1371/journal.pone.0095007 24736445 PMC3988155

[38] Trocoli Torrecilhas AC Tonelli RR Pavanelli WR da Silva JS Schumacher RI de Souza W. Trypanosoma Cruzi: Parasite Shed Vesicles Increase Heart Parasitism and Generate an Intense Inflammatory Response. Microbes Infect (2009) 11:29–39. 10.1016/j.micinf.2008.10.003 19028594

[39] Li Y Liu Y Xiu F Wang J Cong H He S. Characterization of Exosomes Derived From Toxoplasma Gondii and Their Functions in Modulating Immune Responses. Int J Nanomed (2018) 13:467–77. 10.2147/IJN.S151110 PMC5783023 29403276

[40] Wang L Li Z Shen J Liu Z Liang J Wu X. Exosome-Like Vesicles Derived by Schistosoma Japonicum Adult Worms Mediates M1 Type Immune- Activity of Macrophage. Parasitol Res (2015) 114:1865–73. 10.1007/s00436-015-4373-7 25855345

[41] Ahmed SF Oswald IP Caspar P Hieny S Keefer L Sher A. Developmental Differences Determine Larval Susceptibility to Nitric Oxide-Mediated Killing in a Murine Model of Vaccination Against Schistosoma Mansoni. Infect Immun (1997) 65:219–26. 10.1128/iai.65.1.219-226.1997 PMC174579 8975915

[42] Shen J Lai DH Wilson RA Chen YF Wang LF Yu ZL. Nitric Oxide Blocks the Development of the Human Parasite Schistosoma Japonicum. Proc Natl Acad Sci USA (2017) 114:10214–9. 10.1073/pnas.1708578114 PMC5617295 28874579

[43] Zhao Z Zlokovic BV. Remote Control of BBB: A Tale of Exosomes and microRNA. Cell Res (2017) 27:849–50. 10.1038/cr.2017.71 PMC5518990 28674430

[44] Wei J Wu F He A Zeng X Ouyang LS Liu MS. Microglia Activation: One of the Checkpoints in the CNS Inflammation Caused by Angiostrongylus Cantonensis Infection in Rodent Model. Parasitol Res (2015) 114:3247–54. 10.1007/s00436-015-4541-9 26002828

[45] Koo IC Ohol YM Wu P Morisaki JH Cox JS Brown EJ. Role for Lysosomal Enzyme Beta-Hexosaminidase in the Control of Mycobacteria Infection. Proc Natl Acad Sci USA (2008) 105:710–5. 10.1073/pnas.0708110105 PMC2206601 18180457

[46] Schieke SM Phillips D McCoy JP Jr. Aponte AM Shen RF Balaban RS. The Mammalian Target of Rapamycin (mTOR) Pathway Regulates Mitochondrial Oxygen Consumption and Oxidative Capacity. J Biol Chem (2006) 281:27643–52. 10.1074/jbc.M603536200 16847060

[47] Hechtman P Rowlands A. Apparent Hexosaminidase B Deficiency in Two Healthy Members of a Pedigree. Am J Hum Genet (1979) 31:428–38. PMC1685885 484551

[48] Jayaprakash NG Surolia A. Role of Glycosylation in Nucleating Protein Folding and Stability. Biochem J (2017) 474:2333–47. 10.1042/BCJ20170111 28673927

[49] Weichhart T Säemann MD. The Multiple Facets of mTOR in Immunity. Trends Immunol (2009) 30:218–26. 10.1016/j.it.2009.02.002 19362054

[50] Covarrubias AJ Aksoylar HI Yu J Snyder NW Worth AJ Iyer SS. Akt-Mtorc1 Signaling Regulates Acly to Integrate Metabolic Input to Control of Macrophage Activation. eLife (2016) 5:e11612. 10.7554/eLife.11612 26894960 PMC4769166

[51] Hallowell RW Collins SL Craig JM Zhang Y Oh M Illei PB. Mtorc2 Signalling Regulates M2 Macrophage Differentiation in Response to Helminth Infection and Adaptive Thermogenesis. Nat Commun (2017) 8:14208. 10.1038/ncomms14208 28128208 PMC5290163

[52] Maffucci T Cooke FT Foster FM Traer CJ Fry MJ Falasca M. Class II Phosphoinositide 3-Kinase Defines a Novel Signaling Pathway in Cell Migration. J Cell Biol (2005) 169:789–99. 10.1083/jcb.200408005 PMC2171608 15928202

[53] Domin J Gaidarov I Smith ME Keen JH Waterfield MD. The Class II Phosphoinositide 3-Kinase PI3K-C2alpha is Concentrated in the Trans-Golgi Network and Present in Clathrin-Coated Vesicles. J Biol Chem (2000) 275:11943–50. 10.1074/jbc.275.16.11943 10766823

[54] Perez-Riverol Y Csordas A Bai J Bernal-Llinares M Hewapathirana S Kundu DJ. The PRIDE Database and Related Tools and Resources in 2019: Improving Support for Quantification Data. Nucleic Acids Res (2019) 47:D442–d450. 10.1093/nar/gky1106 30395289 PMC6323896

