# Supplementary material for: Exosome-Depleted Excretory-Secretory Products of the Fourth-Stage Larval Angiostrongylus cantonensis Promotes Alternative Activation of Macrophages Through Metabolic Reprogramming by the PI3K-Akt Pathway
Source: Front Immunol. 2021 Jul 23;12:685984. doi: 10.3389/fimmu.2021.685984 (PMC8343011; doi:10.3389/fimmu.2021.685984)
Supplement: Supplementary file 1 [file DataSheet_1.pdf]

Supplementary Material

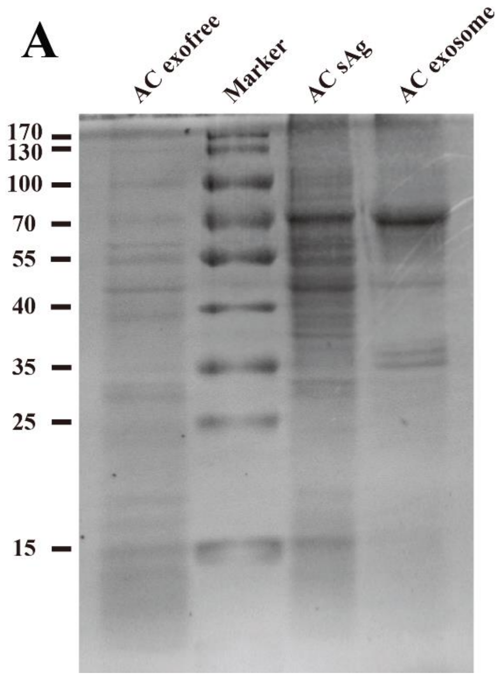

Supplementary Figure 1.

A. Total protein isolated from AC L4 exosome, AC L4 exofree and sAg from AC L4 were measured by SDS-PAGE and stained using silver staining.

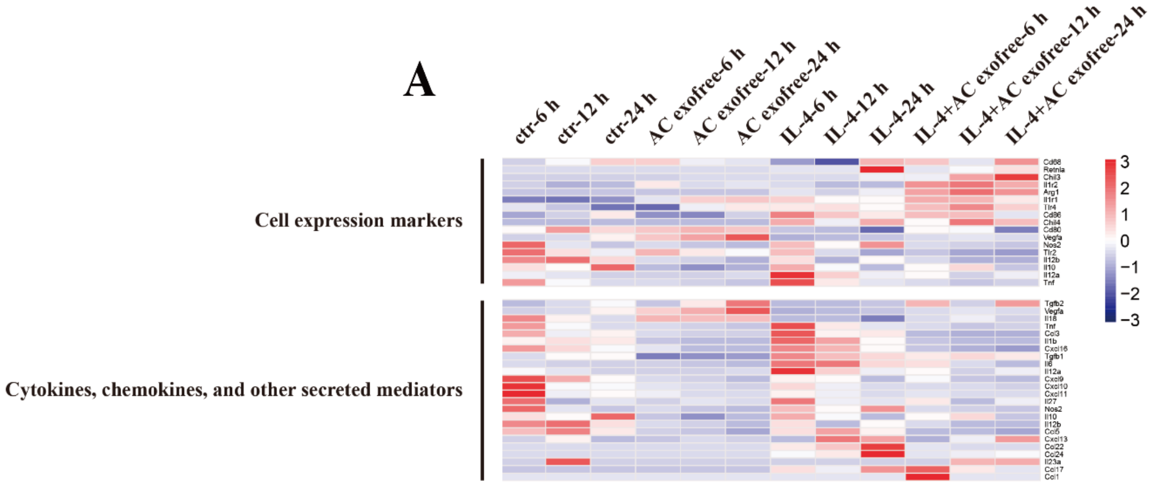

Supplementary Figure 2. Macrophage signature genes based on RNA-seq results.

A. Signature gene expression profile of BMDMs, including macrophage polarization markers, cytokines, chemokines and other secreted mediators. BMDMs were treated with PBS (ctr), free (AC

L4 exofree), IL-4, com (IL-4+AC L4 exofree) for 6h, 12h, and 24 h respectively. The horizontal axis represents genes, and vertical coordinates represents types of treatment.

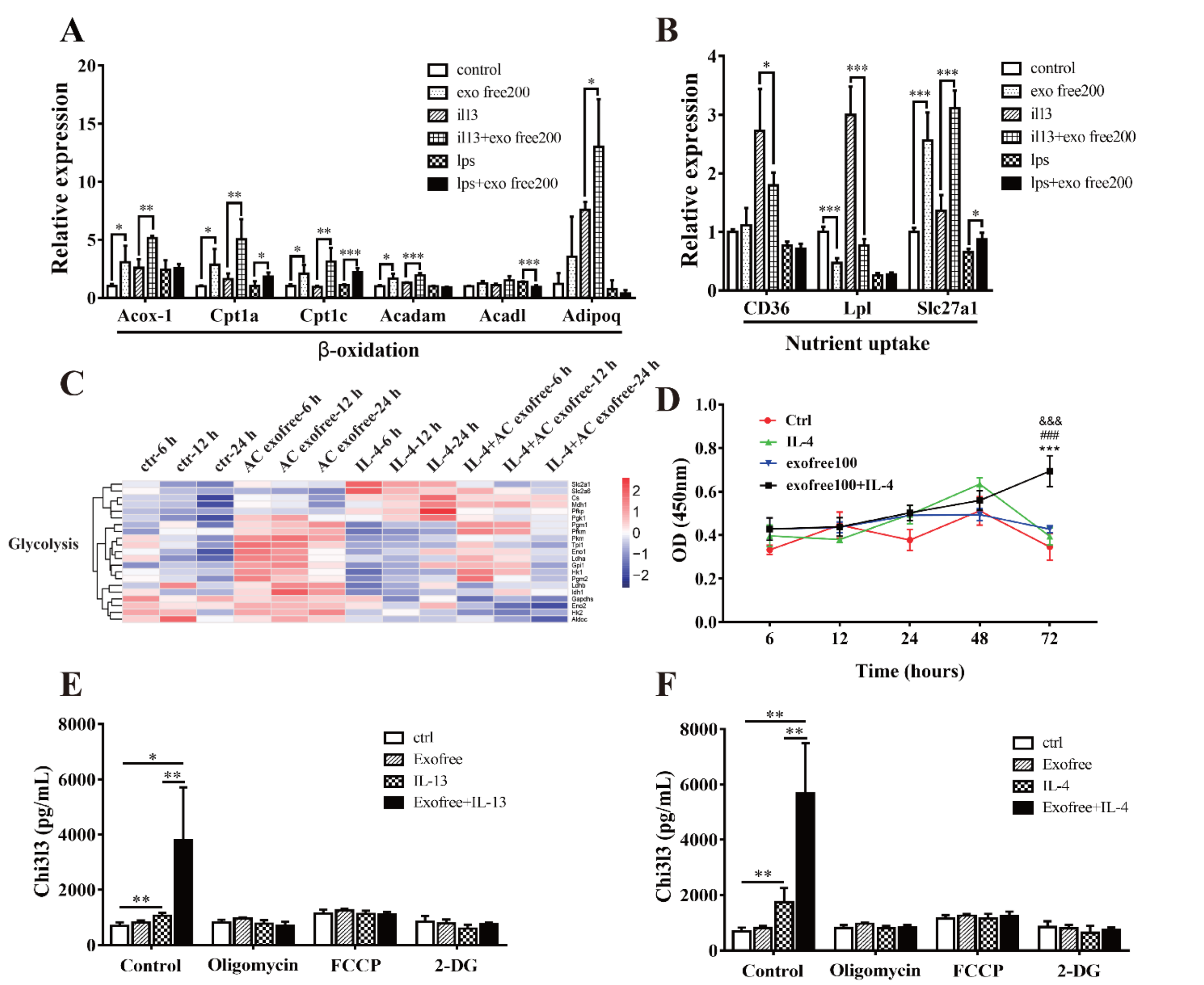

Supplementary Figure 3. Metabolic reprogramming in AC L4 exofree-induced macrophage polarization.

A-B. BMDMs were cultured for 24 h in medium alone or treated with exofree, IL-13, IL-13+exofree, LPS, LPS+exofree, then their mitochondrial function was measured. Relative transcript levels for genes encoding key enzymes in β-oxidation (A) and nutrient uptake (B) are shown. **C.** Gene expression profile of BMDMs related to glycolysis metabolism were shown in a heatmap. BMDMs were treated with PBS (ctr), free (AC L4 exofree), IL-4, com (IL-4+AC L4 exofree) for 6h, 12h, and 24 h respectively. The horizontal axis represents genes, and vertical coordinates represents types of treatment. **D.** Proliferation of BMDMs was determined with CCK8 assay at 6, 12, 24, 48, 72 h after treatment of PBS, IL-4, exofree100, IL-4+exofree100. $^{\&\&\&}P$<0.001, compared with the control group; $^{\#\#\#}P$<0.001, compared with the IL-4 group; ****P*<0.001, compared with the exofree100 group. 100 refer to 100μL AC L4 exofree. **E-F.** BMDMs were treated with PBS, exofree, IL-13, IL-13+exofree **(E)**, and PBS, exofree, IL-4, IL-4+exofree **(F)** in combination of oligomycin, FCCP or 2-

DG for 24 h. The protein levels of Chi3l3 in the culture medium were measured using ELISA. *P<0.05, **P<0.01, ***P<0.001. Data were presented as mean ± SD (Student's t-test).

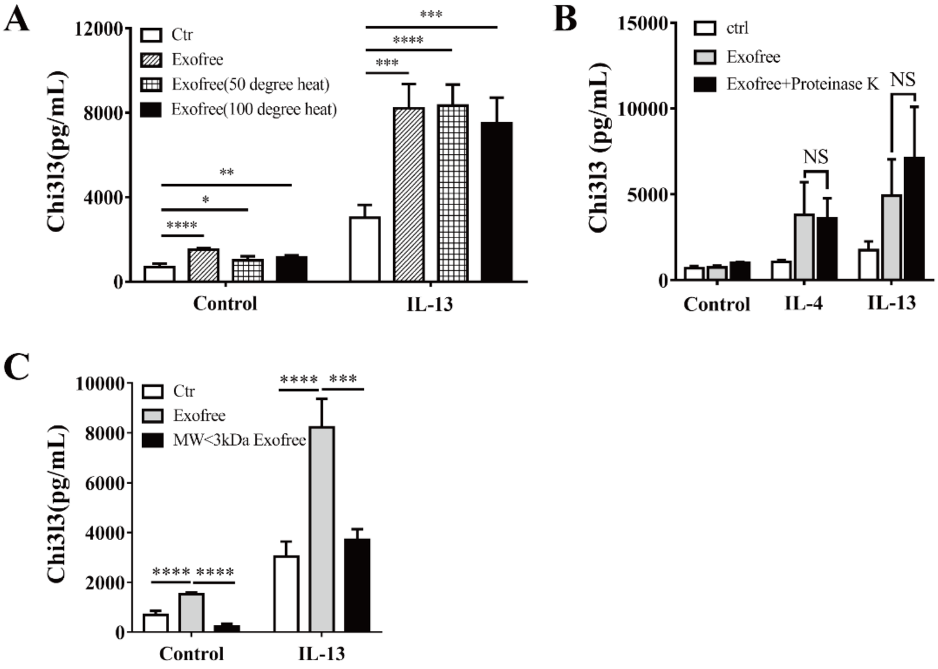

Supplementary Figure 4. Chemical and physical property analysis of AC L4 exofree.

A. Protein expression level of Chi3l3 in the culture medium of BMDMs were measured in presence of PBS, exofree, exofree (50℃ water bath, 15min), exofree (100℃ water bath, 15min), IL-13, IL-13+exosome, IL-13+exosome (50℃ water bath, 15min), IL-13+exosome (100℃ water bath, 15min) for 24 h using ELISA. **B.** Protein expression level of Chi3l3 in the culture medium of BMDMs were measured in presence of PBS, AC exofree, AC exofree (100 µg/mL proteinase K treatment: 58℃ water bath, 2 h; proteinase K inactivation: 100℃ water bath, 15min), IL-13, IL-13+exosome, IL-13+exosome (100 µg/mL proteinase K treatment: 58℃ water bath, 2 h; proteinase K inactivation: 100℃ water bath, 15min) for 24 h using ELISA. **C.** Control (PBS) or AC L4 exofree medium was used unfractionated (whole) or as <3 kDa fraction to stimulate BMDMs as follows. And protein expression level of Chi3l3 in the culture medium of BMDMs were measured 24 h after the stimulation using ELISA. Data information: *P<0.05, **P<0.01, ***P<0.001, ****P<0.0001. Data were presented as mean ± SD (Student's t-test).

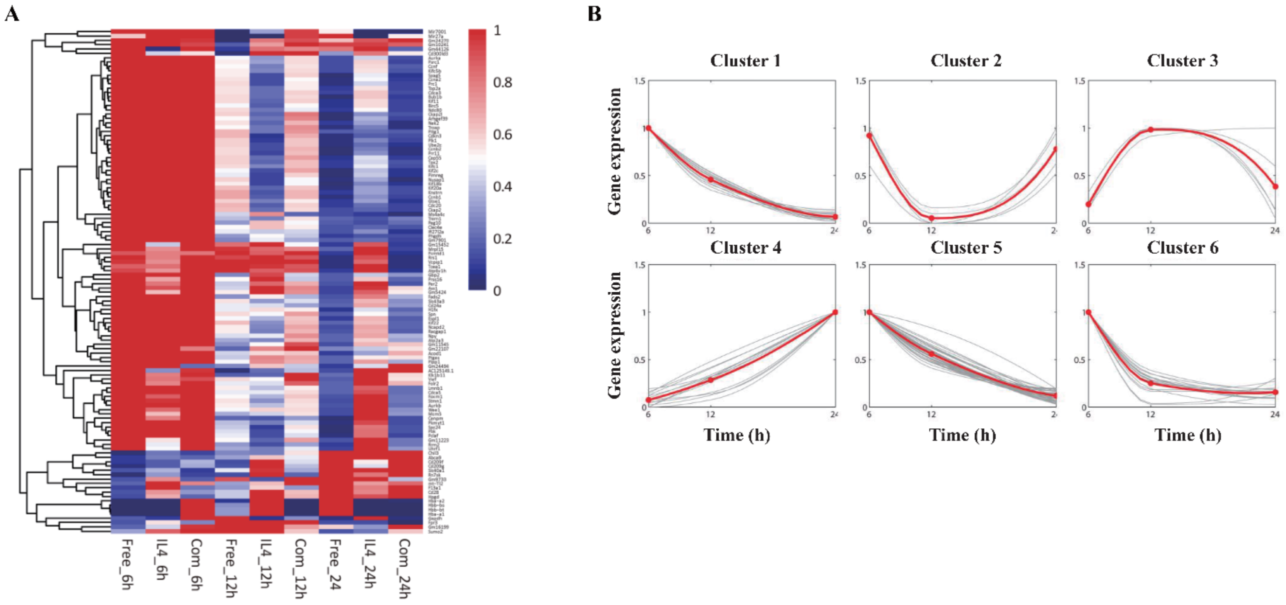

Supplementary Figure 5. A. Expression profile of significant TCGs in BMDMs at 6h, 12h, and 24h post different treatments. The horizontal axis represents genes, and vertical coordinates represents different treatments and time points. Free, IL-4, and Com represent treatments of AC L4 exofree, IL-4, and AC L4 exofree+IL-4 respectively. 6 h, 12 h, and 24 h represent time of the above stimulation. **B.** Expression patterns of TCGs. The x-axis represents time (with the unit as hour), and the y-axis represents the normalized gene expression levels.

A

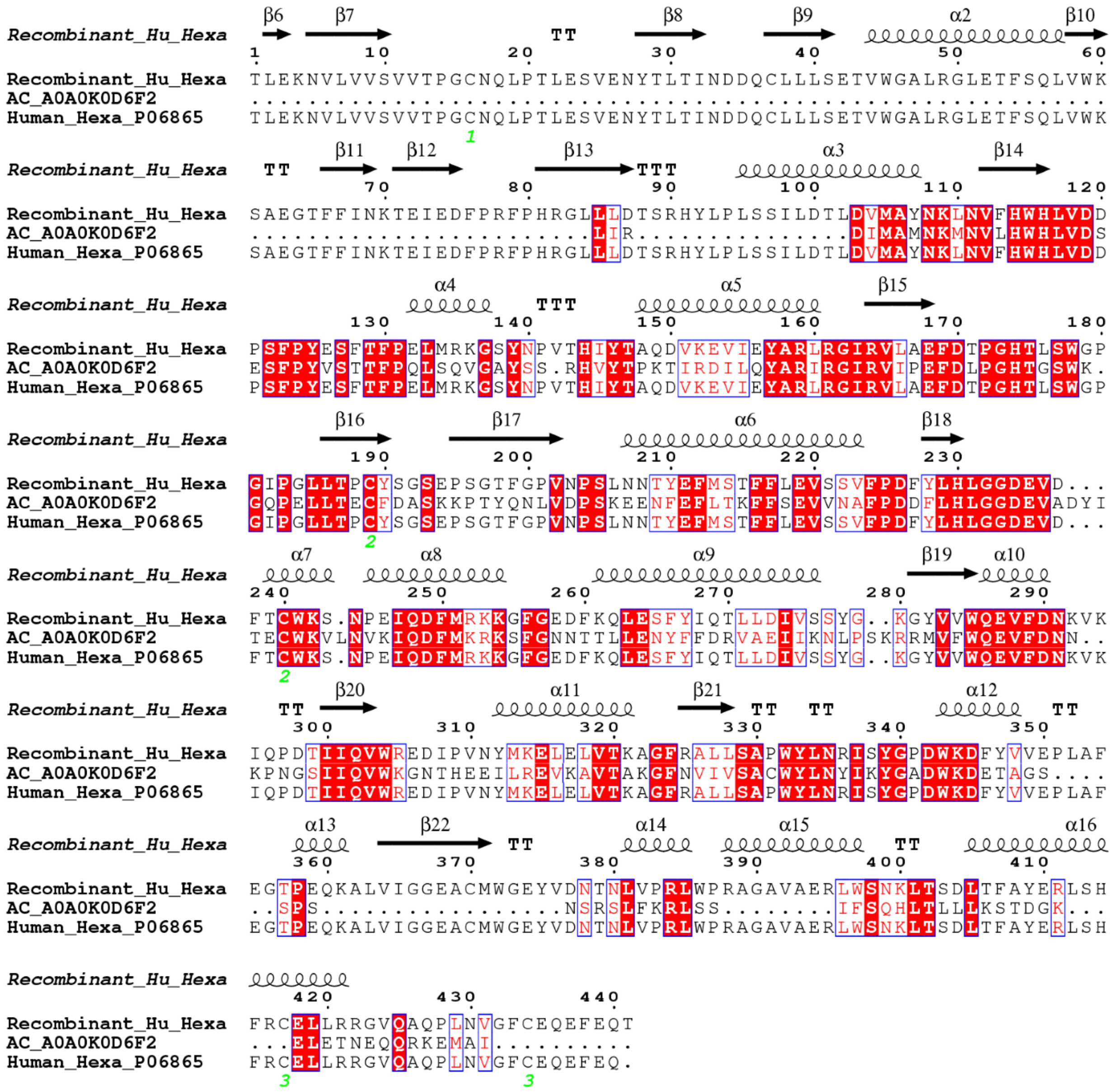

Supplementary Figure 6. A. Multiple sequence alignment of human and AC Hexa protein.

Supplementary Table 1 Sequences of the primers used for qPCR

**Primer**	**Sequence (5'-3')**
m-*Chi3l3*-qPCR -F	CTGAATGAAGGAGCCACTGA
m-*Chi3l3*-qPCR -R	AGCCACTGAGCCTTCAACTT
m-*β-actin*-qPCR -F	GGCATCCTGACCCTGAAGTA
m-*β-actin*-qPCR -R	CTCTCAGCTGTGGTGGTGAA
m-*Arg1*-qPCR-F	CCAGAAGAATGGAAGAGTCAGTGT
m-*Arg1*-qPCR-R	GCAGATATGCAGGGAGTCACC
m-*Nos2*-qPCR-F	CACCAAGCTGAACTTGAGCG
m-*Nos2*-qPCR-R	CGTGGCTTTGGGCTCCTC
m-*Arg1*-qPCR-F	CCAGAAGAATGGAAGAGTCAGTGT
m-*Arg1*-qPCR-R	GCAGATATGCAGGGAGTCACC
m-*IL-12b*-qPCR-F	GGACATCATCAAACCAGACC
m-*IL-12b*-qPCR-R	GAATTGTAATAGCGATCCTGAG
m-*IL-1b*-qPCR-F	TTCAGGCAGGCAGTATCACTC
m-*IL-1b*-qPCR-R	GAAGGTCCACGGGAAAGACAC
m-*IL-6*-qPCR-F	GTTGCCTTCTTGGGACTGATG
m-*IL-6*-qPCR-R	GGGAGTGGTATCCTCTGTGAAGTCT
m-*IL-10*-qPCR-F	AGCCTTATCGGAAATGATCCAGT
m-*IL-10*-qPCR-R	GGCCTTGTAGACACCTTGGT

m-*Acox1*-qPCR-F	GCCAGGACTATCGCATGATT
m-*Acox1*-qPCR-R	GCCCAACTGTGACTTCCATC
m-*Cpt1b*-qPCR-F	CCAGACCCATACACCGACAG
m-*Cpt1b*-qPCR-R	GTCTCAGAGCCTCCCGACTA
m-*Cpt1c*-qPCR-F	TCTTCACTGAGTTCCGATGGG
m-*Cpt1c*-qPCR-R	ACGCCAGAGATGCCTTTTCC
m-*Acadm*-qPCR-F	AGGGTTTAGTTTTGAGTTGACGG
m-*Acadm*-qPCR-R	CCCCGCTTTTGTCATATTCCG
m-*Acadl*-qPCR-F	CTTTTCCTCGGAGCATGACA
m-*Acadl*-qPCR-R	GACCTCTCTACTCACTTCTCCAG
m-*Adipoq*-qPCR-F	TGTTCCTCTTAATCCTGCCCA
m-*Adipoq*-qPCR-R	CCAACCTGCACAAGTTCCCTT
m-*CD36*-qPCR-F	ATGGGCTGTGATCGGAACTG
m-*CD36*-qPCR-R	GTCTTCTCAATAAGCATGTCTCC
m-*Lpl*-qPCR-F	GGGAGTTTGGCTCCAGAGTTT
m-*Lpl*-qPCR-R	TGTGTCTTCAGGGGTCCTTAG
m-*slc27a1*-qPCR-F	CGCTTTCTGCGTATCGTCTG
m-*slc27a1*-qPCR-R	GATGCACGGGATCGTGTCT

Supplementary Table 2 Summary table of the treatments and the time of experiment in Figure 3 L and Figure 3M.

BMDMs were pretreated in 0~24h as shown, the medium was discarded at 24h. Then, BMDMs were washed with PBS for three times, and restimulated as shown below. Finally, the 24~48h-BMDMs culture medium was collected for Chi3l3 ELISA analysis.

Group serial number	0-24 h stimulation	Wash step	24-48 h stimulation
1	PBS	wash with PBS for 3 times	PBS
2	PBS	wash with PBS for 3 times	exofree
3	PBS	wash with PBS for 3 times	IL-4
4	PBS	wash with PBS for 3 times	exofree+IL-4
5	exofree	wash with PBS for 3 times	PBS
6	exofree	wash with PBS for 3 times	exofree
7	exofree	wash with PBS for 3 times	IL-4
8	exofree	wash with PBS for 3 times	exofree+IL-4
9	IL-4	wash with PBS for 3 times	PBS
10	IL-4	wash with PBS for 3 times	exofree
11	IL-4	wash with PBS for 3 times	IL-4
12	IL-4	wash with PBS for 3 times	exofree+IL-4
13	exofree+IL-4	wash with PBS for 3 times	PBS
14	exofree+IL-4	wash with PBS for 3 times	exofree
15	exofree+IL-4	wash with PBS for 3 times	IL-4
16	exofree+IL-4	wash with PBS for 3 times	exofree+IL-4
